# The burden of diabetes mellitus and impaired fasting glucose in an urban population of Sri Lanka

**DOI:** 10.1111/dme.12013

**Published:** 2013-02-20

**Authors:** M J Pinidiyapathirage, A Kasturiratne, U K Ranawaka, D Gunasekara, N Wijekoon, K Medagoda, S Perera, F Takeuchi, N Kato, T Warnakulasuriya, A R Wickremasinghe

**Affiliations:** 1 Faculty of Medicine, University of KelaniyaRagama, Sri Lanka; 2 Department of Gene Diagnostics and Therapeutics, Research Institute, National Center for Global Health and MedicineTokyo, Japan; 3 Ministry of HealthSri Lanka

## Abstract

**Aims:**

To describe the burden of diabetes mellitus and impaired fasting glucose in middle-aged residents (35–64 years) in an urban area of Sri Lanka.

**Methods:**

A cross-sectional survey was conducted in the Ragama Medical Officer of Health area, from which 2986 participants (1349 men and 1637 women) were randomly selected from the electoral registry between January and December 2007. The participants underwent a physical examination and had their height, weight, waist and hip circumferences and blood pressure measured by trained personnel. Fasting blood samples were taken for measurement of glucose, HbA_1c_ and lipids. The prevalence of diabetes (fasting plasma glucose > 7 mmol/l) and impaired fasting glycaemia (fasting plasma glucose 5.6–6.9 mmol/l) and major predictors of diabetes in Sri Lanka were estimated from the population-based data.

**Results:**

Age-adjusted prevalence of diabetes mellitus in this urban population was 20.3% in men and 19.8% in women. Through the present screening, 263 patients with diabetes and 1262 with impaired fasting glucose levels were identified. The prevalence of newly detected diabetes was 35.7% of all patients with diabetes. Among patients with diabetes, only 23.8% were optimally controlled. In the regression models, high BMI, high waist circumference, high blood pressure and hypercholesterolaemia increased the fasting plasma glucose concentration, independent of age, sex and a family history of diabetes.

**Conclusions:**

Our data demonstrate the heavy burden of diabetes in this urban population. Short- and long-term control strategies are required, not only for optimal therapy among those affected, but also for nationwide primary prevention of diabetes.

## Introduction

Diabetes mellitus, which was once considered a disease of the West and the affluent, is now rising at an alarming rate, affecting all populations worldwide. The International Diabetes Federation (IDF) estimated the worldwide prevalence of diabetes mellitus to be 366 million in 2011, with projections that the prevalence will increase to 552 million by 2030 1.

With a global shift in the populations affected with diabetes, South-East Asia has become an emerging epicentre of this chronic disease. According to the International Diabetes Federation, one fifth of all adults with diabetes in the world live in the South-East Asia Region. In 2011, 8.3% of the adult population, or 71.4 million South Asians were estimated to have diabetes. The projections depict that the number of people with diabetes in the region will increase to 120.9 million by 2030; i.e. 10.2% of the adult population 2.

Sri Lanka is currently experiencing an epidemic of diabetes mellitus as a result of the epidemiologic and demographic transitions that have interacted with lifestyle changes. Non-communicable diseases constitute the leading cause of death in the country and the leading cause of hospitalization in government hospitals throughout the country 3. The life expectancy of Sri Lankans has been increasing steadily since the 1950s and 1960s 4.

The estimated prevalence of diabetes mellitus has increased from 2.5% in 1993 to 8.5% in 2004 5. In 2005, prevalences of 14.2 and 13.5% of diabetes were reported in men and women, respectively 6. Surveys have reported high risk factor prevalence in the community. We report the burden of diabetes in an urban/suburban community resident in the Ragama Medical Officer of Health area in Sri Lanka based on a survey carried out in 2007.

## Patients and methods

The Ragama Medical Officer of Health area, an urban/suburban area comprising a diverse population, is located in the Gampaha district of the Western Province of Sri Lanka, 20 km from the capital city of Colombo. It extends over 25 km^2^ and the estimated mid-year population in 2007 was 75 591. The Colombo North Teaching Hospital, the main teaching hospital of the Faculty of Medicine, University of Kelaniya, is situated in the Ragama Medical Officer of Health area. The study was conducted from January 2007 to September 2007.

### Study population

Our sample comprised persons between 35 and 64 years of age. The sampling frame for the study was developed using the electoral register of 2006 maintained by 21 Grama Niladharis (the government administrative officers of the smallest geographic administrative unit) of the Ragama Medical Officer of Health area by extracting the name, age and sex of individuals born before 1972. The population was then stratified into three age groups (35–44, 45–54 and 55–64 years) and a list of all individuals in each stratum was prepared. A random sample of 200 individuals was obtained from each Grama Niladhari area using random numbers generated by the statistical program, PEPI v.4.0 (http://www.simtel.net/pub/pd/54632.html), in the following ratio: 40 persons between 35 and 44 years (sampling fraction = 0.073), 80 persons between 45 and 54 years (sampling fraction = 0.183) and 80 persons between 55 and 64 years (sampling fraction = 0.304). All Grama Niladhari areas had almost equal populations. All selected individuals were invited to participate in the study. In total, 3012 persons (response rate of 72%) participated in the study. The highest response rate (79%) was observed in the oldest age group (55–64 years). The lowest response rate (61%) was observed in the 35- to 44-year age group and, in the 45- to 54-year age group, the response rate was 68%. Data analysis was performed for 2986 individuals for whom complete data were available.

Each participant was assigned an individual identification number. Subjects were invited to participate in the study by letter and an appointment was given at the faculty clinic for history taking, examination and investigations; written instructions were given on when to stop consuming food.

At the clinic, the study was explained in detail to all participants and voluntary written consent obtained. A detailed history was taken by trained interviewers to obtain information on socio-demographic characteristics, current medical history, past medical history, family history, risk factors, dietary and physical activity patterns, and the reproductive history in women. Participants were subjected to a physical examination and height, weight, blood pressure 7 and waist and hip circumferences were measured. Ten millilitres of venous blood was drawn after a 14-h fast for assay of lipid profiles, fasting plasma glucose and HbA_1c_. If the participant had not been fasting for at least 12 h, a new appointment was given for blood tests.

The collected blood samples were centrifuged and separated immediately. The samples were transported to the Nawaloka Metropolis Clinical Laboratory, an accredited laboratory in Sri Lanka with ISO 9002 certification and quality controlled by Randox Laboratory UK, for analysis. Subjects detected to have any disorders were followed up.

Smoking status was classified as never smoker, ex-smoker and current smoker. Alcohol consumption was categorized as never consumed, ex-consumer, current (infrequent) consumer and current (regular) consumer. An ex-consumer of alcohol should have abstained from alcohol for at least the three preceding months. A current (infrequent) alcohol consumer was defined as a person who consumes alcohol less than once a week irrespective of the quantity. A current (regular) consumer was defined as a person who consumes alcohol at least once a week.

An International Physical Activity Questionnaire (IPAQ) that was culturally adapted and validated by triangulation was used to assess physical activity 8. Participants' activity levels were classified as ‘low’, ‘moderate’ or ‘high’ based on scores of their overall level of activity, which required summation of the duration (in min) and frequency (days) of vigorous and moderate intensity physical activities and walking by each participant in relation to occupation, transportation, housework, recreation, sports and leisure time activities during the previous week.

Hyperglycaemia was defined as a fasting plasma glucose level ≥ 7 mmol/l; impaired fasting glycaemia as a fasting plasma glucose level from 5.6 to 6.9 mmol/l 9. Hypertension was defined as a systolic blood pressure ≥ 140 mmHg or a diastolic blood pressure ≥ 90 mmHg. Dyslipidaemia was defined as a total cholesterol level ≥ 6.2 mmol/l or a serum LDL cholesterol level ≥ 4.1 mmol/l or a serum HDL cholesterol level < 1 mmol/l for men and < 1.3 mmol/l for women or a serum triglyceride level ≥ 2.24 mmol/l. Obesity was defined as a BMI ≥ 25 kg/m^2^ for both men and women. Central obesity was defined as waist circumferences ≥ 90 cm for men and ≥ 80 cm for women.

### Data analysis

All data were entered into an Epi Info database (Centers for Disease Control and Prevention, Atlanta, GA, USA). Frequency distributions, descriptive statistics and point estimates for prevalence were generated. The significance of differences between means was tested using Student's *t*-test. Regression models were developed, with fasting plasma glucose as the dependent variable, with each of the following as independent variables separately adjusted for age and sex: family history, smoking, high BMI, high waist circumference, low physical activity, high blood pressure and hypercholesterolaemia. Similar models were developed controlling for age, sex and family history of diabetes mellitus, and age, sex, family history of diabetes mellitus and smoking. A total of 18 models are presented. All those with a past history of diabetes or categorized as having diabetes on screening were excluded from the regression analysis. SPSS version 16.0 (SPSS Inc., Chicago, IL, USA) was used for data analysis.

## Results

The total number of subjects was 2986 (1349 men and 1637 women), with 515 in the 35- to 44-year age group, 1140 in the 45- to 54-year age group and 1331 in the 55- to 64-year age group. The majority of participants were Sinhalese and Buddhist. The prevalence of current smoking and alcohol consumption was negligible among women. Among men, the prevalence of current smoking was 36.0% and of current alcohol consumption was 25.1%.

**FIGURE 1:**
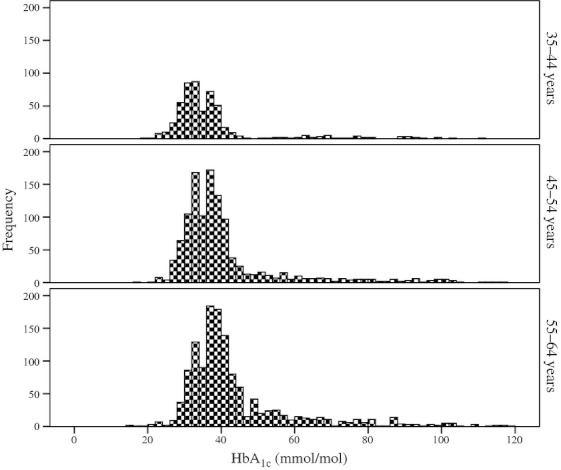
Distribution of HbA_1c_ among study participants without a past history of diabetes mellitus.

Based on fasting plasma glucose levels (excluding previously diagnosed persons with diabetes), approximately 9.4% of the population had fasting plasma glucose levels above ≥ 7 mmol/l and 50.3% of the population had fasting plasma glucose levels between 5.6 and 6.9 mmol/l (Table 1). Based on the criteria of a fasting blood glucose ≥ 7 mmol/l and/or an HbA_1c_ ≥ 48 mmol/mol (6.5%), 10.5% of those without previously diagnosed diabetes were detected as having diabetes. The full histogram of the distribution of HbA_1c_ among those without a history of diabetes is given in Fig. 1. Among those without a history of diabetes, prevalence of hyperglycaemia increased with age; women had higher prevalence of hyperglycaemia than men in the older age groups. The percentage of women having impaired plasma glucose levels was less than that of men in all age groups (Table 1). Table 2 gives the prevalence of diabetes (those with a history of diabetes and newly diagnosed) by age groups. Prevalence of diabetes increased with age in both sexes. Age-adjusted prevalence of diabetes for men was 20.3% and 19.8% for women (Table 2).

**Table 1:** Glycaemic status of participants without a history of diabetes by age group

	% men (95% CI)			% women (95% CI)			
			
Measurement	35–44 years (*n* = 217)	45–54 years (*n* = 441)	55–64 years (*n* = 497)	35–44 years (*n* = 258)	45–54 years (*n* = 542)	55–64 years (*n* = 556)	Total% (95% CI) (*n* = 2511)
Based on fasting plasma glucose*							
Hyperglycaemia ≥ 7 mmol/l	6.0 (2.8–9.1)	9.3 (6.6–12.0)	10.3 (7.6–12.9)	3.5 (1.3–5.7)	10.5 (7.9–13.0)	11.5 (8.8–14.1)	9.4 (8.3–10.5)
Impaired fasting plasma glucose5.5–6.9 mmol/l	46.5 (39.8–53.1)	46.3 (41.6–50.9)	51.9 (47.5–56.2)	36.4 (30.5–42.2)	43.5 (39.3–47.7)	50.4 (46.2–54.6)	50.3 (48.3–52.3)
Normal < 5.5 mmol/l	47.5 (40.8–54.1)	44.4 (39.8–49.0)	37.8 (33.5–42.1)	60.1 (54.1–66.1)	45.9 (41.7–50.0)	38.1 (34.1–42.1)	40.4 (38.5–42.3)
Based on HbA_1c_†							
≥ 48 mmol/mol (≥ 6.5%)	11.6 (7.3–15.9)	15.0 (11.6–18.3)	19.6 (16.1–23.1)	7.3 (4.1–10.5)	15.6 (12.5–18.6)	25.7 (22.1–29.3)	17.7 (16.2–19.1)
42–47 mmol/mol (6.0–6.4%)	2.5 (0.4–4.6)	6.8 (4.4–9.1)	10.4 (9.6–11.2)	2.9 (0.85–4.9)	6.6 (4.5–8.7)	12.7 (9.9–15.5)	8.2 (7.12–9.3)
< 42 mmol/mol (< 6.0%)	85.9 (81.3–90.5)	78.2 (74.3–82.0)	70.0 (66.0–74.0)	89.8 (86.1–93.5)	77.8 (74.3–81.3)	61.5 (57.5–65.5)	74 (72.2–75.7)

+Fasting plasma glucose measurement was not available for one woman.

† HbA_1c_ measurement was not available in one woman and one man.

**Table 2:** Prevalence of diabetes mellitus in the study population

	Men		Women*	
		
Age group (years)	*n*	% (95% CI)	*n*	% (95% CI)
35–44	241	15.8 (11.6–20.8)	274	9.5 (6.4–13.4)
45–54	501	21.2 (17.7–24.9)	639	24.6 (21.3–28.0)
55–64	607	28.0 (24.5–31.7)	723	33.1 (29.7–36.6)
35–64†	1349	20.3 (18.2–22.5)	1636	19.8 (17.9–21.7)

* Fasting plasma glucose and HbA_1c_ measurements were not available for one woman.

† Age adjusted to the population structure of the district (Gampaha district).

**FIGURE 2:**
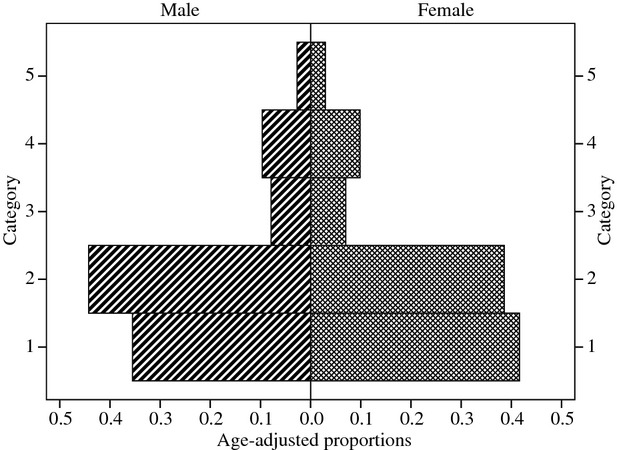
Diabetes pyramid of an urban Sri Lankan population: category 1, normal glycaemia; category 2, impaired fasting glucose; category 3, newly detected (at screening); category 4, patients with diabetes—without suboptimal control; category 5, patients with diabetes—with optimal control.

Of all patients with diabetes in the study population [those with a past history of diabetes and newly detected at screening based on the criteria of fasting blood glucose ≥ 7 mmol/l and/or an HbA_1c_ ≥ 48 mmol/mol (6.5%)], 35.7% were previously undiagnosed. Using an HbA_1c_ < 48 mmol/mol (6.5%) as the cut-off for optimal control of diabetes, only 23.8% of previously diagnosed patients with diabetes (24.7% of men and 23.2% of women) were optimally controlled. When persons with a history of diabetes were excluded, 47% of the study population was detected as having impaired fasting glucose. Figure 2 gives the age-adjusted proportions of persons with normal glycaemia, impaired fasting glucose, newly detected diabetes (at screening), history of diabetes without suboptimal control and history of diabetes with optimal control.

Patients with diabetes had significantly higher waist circumferences, systolic blood pressure and serum triglyceride concentrations and significantly lower LDL cholesterol concentrations as compared with those without diabetes among both men and women (Table 3).

**Table 3:** Risk factors by diabetes status and sex

	Men			Women		
				
Variable	Mean (sd) among subjects with diabetes *n* = 299	Mean (sd) among subjects without diabetes *n* = 1050	*P*	Mean (sd) among subjects with diabetes *n* = 410	Mean (sd) among subjects without diabetes *n* = 1227	*P*
BMI (kg/m^2^)	24.3 (3.8)	22.9 (3.9)	< 0.001	25.1 (3.5)	24.9 (4.4)	0.346
Waist circumference (cm)	90.4 (9.8)	85.3 (10.3)	< 0.001	87.9 (8.9)	84.8 (11.3)	< 0.001
Systolic blood pressure (mmHg)	138.4 (21.9)	133.2 (20.9)	0.002	141.9 (21.7)	135.0 (22.5)	< 0.001
Diastolic blood pressure (mmHg)	81.2 (12.6)	78.9 (12.6)	0.018	80.5 (10.1)	79.5 (12.3)	0.224
Total cholesterol (mmol/l)	5.1 (1.0)	5.3 (1.1)	0.053	5.5 (1.2)	5.6 (1.1)	0.200
HDL cholesterol (mmol/l)	1.2 (0.1)	1.3 (0.1)	0.029	1.3 (0.1)	1.3 (0.1)	0.194
LDL cholesterol (mmol/l)	3.1 (0.8)	3.3 (0.9)	0.001	3.5 (1.1)	3.7 (1.0)	< 0.001
Triglycerides (mmol/l)	1.8 (0.9)	1.5 (0.8)	< 0.001	1.7 (0.9)	1.4 (0.75)	< 0.001

sd, standard deviation.

In the regression models of fasting plasma glucose adjusted for age and sex, all variables considered separately, except current smoking, increased the concentration of fasting plasma glucose. After adjusting for age, sex and a family history of diabetes mellitus, high BMI, high waist circumference, high blood pressure and hypercholesterolaemia when considered separately, significantly increased fasting plasma glucose concentration. Addition of smoking to the variables adjusted for did not change the direction or the magnitude of the fasting plasma glucose associations with high BMI, high waist circumference, high blood pressure and hypercholesterolaemia (Table 4).

**Table 4:** Regression models with fasting blood glucose as the dependent variable adjusted for different variables

Variables adjusted for	Variable*	Regression coefficient of variable in column 2 after adjusting for variables in column 1†	95% CI of regression coefficient	*P*-value
Age and sex	Family history	0.110	0.06–0.159	< 0.001
	Smoking	−0.067	−0.134 to 0.000	0.048
	BMI > 25 kg/m^2^	0.164	0.117–0.211	< 0.001
	High waist circumference	0.168	0.120–0.215	< 0.001
	Low physical activity	0.063	0.004–0.123	0.036
	High blood pressure	0.168	0.120–0.216	< 0.001
	Hypercholesterolaemia	0.112	0.066–0.158	< 0.001
Age, sex and family history of diabetes mellitus	Smoking	−0.062	−0.129 to 0.004	0.065
	BMI > 25 kg/m^2^	0.157	0.110–0.204	< 0.001
	High waist circumference	0.160	0.112–0.207	< 0.001
	Low physical activity	0.059	0.000–0.118	0.052
	High blood pressure	0.164	0.116–0.212	< 0.001
	Hypercholesterolaemia	0.106	0.061–0.152	< 0.001
Age, sex, family history and smoking	BMI > 25 kg/m^2^	0.155	0.108–0.202	< 0.001
	High waist circumference	0.159	0.111–0.206	< 0.001
	Low physical activity	0.058	0.000–0.117	0.053
	High blood pressure	0.162	0.114–0.210	< 0.001
	Hypercholesterolaemia	0.106	0.060–0.152	< 0.001

* Each row of the table represents a separate regression model.

† Regression coefficients adjusted only for the variables in column 1.

## Discussion

We report an age-adjusted prevalence of diabetes of 20% in this population, which is higher than that reported in similar populations for both men and women within the country or in the region 2,6,10–13. In 2005, Wijewardena *et al*. reported prevalences of 14.2 and 13.5% in 30- to 65-year-old men and women from five provinces of Sri Lanka 6. Katulanda *et al*. (in 2006) estimated the age- and sex-standardized prevalence of diabetes as 16.4% for the urban adult Sri Lankan population over 20 years based on the 75-g oral glucose tolerance test 10. Ramachandran *et al*. reported an age-standardized prevalence of diabetes of 12.1% in a study conducted in six major cities covering all regions of India 11. In urban areas of Bangladesh, the prevalence of diabetes was estimated to be 8.0% 12. In urban Pakistan, the prevalence of diabetes was 6% 13. In Nepal, the prevalences of diabetes in urban and rural areas were estimated to be 15 and 3%, respectively 14. Our study, being the most recent and comprising an older population from an urban area, may explain the differences in the estimates between the studies. These results may also be a reflection of the epidemiologic transition that Sri Lanka has experienced earlier than other countries in the South Asian region and the increase in the number of patients with diabetes projected in the region by 2030 by the International Diabetes Federation 2. The prevalence of diabetes we report is likely to be an underestimate of the actual prevalence, as fasting plasma glucose levels were used in the classification of subjects with diabetes in this study instead of the 2-h plasma glucose test that is considered to be more sensitive to diagnose diabetes, especially in older populations 15.

Of the 474 subjects who had a history of diabetes, all were on treatment except for nine men and nine women. Optimal glycaemic control, defined as a HbA_1c_ < 48 mmol/mol (6.5%), was achieved in only 48 men (24.7%) and 65 women (23.2%), highlighting the need for good management of patients. Although national best practice guidelines for the treatment of diabetes are available 16, they are not adhered to on a routine basis. In Sri Lanka, the government provides free healthcare services and medication. Sometimes there are shortages of drugs in government hospitals and patients have to purchase these drugs from the private sector as out-of-pocket expenses. In government hospitals, HbA_1c_ assays are not routinely performed. As some patients find it difficult to afford these tests from the private sector, there is a reluctance of physicians to order them.

In our study, there were 121 men and 142 women who had undetected diabetes, fasting blood glucose ≥ 7 mmol/l and/or an HbA_1c_ ≥ 48 mmol/mol (6.5%), comprising one third of all patients with diabetes (35.7%) in the community. Katulanda *et al*. reported similar results; i.e. that 36% of all patients with diabetes were undetected 10. Impaired fasting glycaemia was prevalent in 47.5% of men and 43.9% of women (45.5% of the total population). Wijewardena *et al*., using fasting plasma glucose of between 6.1 and 7 mmol/l as the cut-off, reported impaired fasting glycaemia in 14.2% of the men and 14.1% of the women in their study 6. Katulanda *et al*. reported a pre-diabetes prevalence of 11.5% (13.6% for men and 11.0% for women) in the adult Sri Lankan population over 20 years of age 10; pre-diabetes was defined as impaired fasting glucose or impaired glucose tolerance or both. The high prevalence of impaired fasting glucose we report is probably attributable to our study population being an older urban population. Selecting an older urban adult population for our study was deliberate. Chronic diseases such as diabetes mellitus are more likely to emerge for the first time in this age group. Being urban would increase its prevalence. We are consistently following up this cohort for any siginificant outcome events and the study team provides long-term medical care for those who are diagnosed as having any chronic disease condition. The size of the study population and its geographical distribution make it possible to provide follow-up care even under low-resource settings. Katulanda *et al*. in their study have covered a large geographic proportion of the country, both urban and rural, but the study was not intended to follow up the participants after their initial investigations.

The prevalence of cardiovascular disease risk factors was higher in patients with diabetes as compared with those without diabetes among both men and women. Katulanda *et al*. reported similar findings 10. In the regression models, high BMI, high waist circumference, high blood pressure and hypercholesterolaemia significantly increased fasting plasma glucose concentration after adjusting for age, sex, family history of diabetes and smoking. All the above risk factors are modifiable risk factors; hence, it is important that individuals with these risk factors be identified early and necessary preventive strategies be implemented.

The burden of impaired glycaemia and diabetes in this population in absolute terms is a dilemma for public health professionals in designing and implementing control programmes. Although the sample in this study is from a restricted geographic area, which may be considered a limitation, our findings are similar to those reported from other studies of urban areas within the country and the region. The large number of known patients with diabetes with suboptimal control implies that even those identified as having diabetes do not have access to proper management, or that even those who do have access do not receive optimal clinical care. In the short term, the major considerations should focus on identifying patients with diabetes and providing them with optimal treatment. A concerted effort should be made to target effective interventions to the population with impaired fasting glucose, primarily aimed at modifying lifestyles. In the long term, health promotion activities to increase knowledge of, and to improve attitudes towards, diabetes should be focused on a mass scale starting at an early age. In Sri Lanka, screening programmes have commenced and a few intervention options are currently being field tested. Challenges in developing such strategies include identifying an effective delivery mechanism, especially when such programmes were non-existent.
